# Self-reported vs. objectively assessed adherence to inhaled corticosteroids in asthma

**DOI:** 10.1186/s40733-021-00072-2

**Published:** 2021-05-31

**Authors:** Frodi Fridason Jensen, Kjell E. J. Håkansson, Britt Overgaard Nielsen, Ulla Møller Weinreich, Charlotte Suppli Ulrik

**Affiliations:** 1 Department of Respiratory Medicine, Copenhagen University Hospital - Hvidovre, Hvidovre, Denmark; 2 Department of Respiratory Diseases, Aalborg University Hospital, Aalborg, Denmark; 3 The Clinical Institute, Aalborg University, Aalborg, Denmark; 4 Institute of Clinical Medicine, University of Copenhagen, Copenhagen, Denmark

**Keywords:** Foster score, Medication possession ratio, Controller medication, Asthma, Self-assessed adherence, Patient-reported outcome

## Abstract

**Background:**

Adherence to inhaled corticosteroids (ICS) in asthma is vital for disease control. However, obtaining reliable and clinically useful measures of adherence remains a major challenge. We investigated the association between patient-reported adherence and objectively measured adherence based on filled prescriptions with inhaled corticosteroids in adults with asthma.

**Methods:**

In total, 178 patients with asthma were asked to self-assess adherence during routine visits at a respiratory outpatient clinic. Self-assessment was performed using Foster score (“*How many days in a 7-day week do you take your medication as prescribed?*”, with the answer divided by 7). Objective adherence was calculated as medication possession ratio (MPR). Bivariate and multivariable linear regression, adjusted for age, sex, FEV_1_, GINA treatment step, excessive use of SABA, and history of exacerbations were used for analyses.

**Results:**

Of the included patients, 87.6% reported a Foster score of 100%, while the mean ICS MPR was 54.0% (SD 25%). Complex regimens such as twice-daily dosing or dual inhaler-use were associated with lower adherence (*p* = 0.015 and *p* < 0.001, respectively).

Foster score was predictive of ICS MPR, with an absolute 32% increase in MPR between patients reporting Foster scores of 0 and 100% (95% CI 13–50%, *p* < 0.001). Female sex predicted higher ICS MPR (*p* = 0.019). Previous asthma-related hospitalization(s) predicted lower ICS MPR (*p* = 0.039).

**Conclusion:**

Although a weak association was found between Foster score and ICS MPR, findings do not support the use of Foster score, and by that self-reported adherence, as a reliable marker of controller adherence in asthma due to significant mismatch between patient-reported adherence and MPR. Future studies should address the complex interplay between patient-reported and objectively assessed adherence to controller medication in asthma.

**Supplementary Information:**

The online version contains supplementary material available at 10.1186/s40733-021-00072-2.

## Quick look

### Current knowledge

Assessing adherence to ICS is central to asthma care with a plethora of assessment methods, yet a lack of standardization in clinical use. A verified, reliable patient-assessed method with acceptable correlation to objective measurements of adherence is yet to be established. In Danish national guidelines, the one-item questionnaire Foster score is recommended for use in adherence assessment without a clear correlation to clinical outcomes.

### What this paper contributes to our knowledge

Our findings suggest that the routinely used, patient-reported Foster score correlates poorly to objective measures of adherence when used in clinical practice. Furthermore, it builds on current evidence that objective, clinical parameters are insufficient to explain patterns of adherence. Furthermore, subjective factors and treatment regimens are important elements in asthma controller adherence and needs to be individually addressed beyond the use of simple scores.

## Introduction

Asthma is one of the most common chronic diseases and impacts patients throughout their lives [1–3]. With appropriate diagnostic workup and pharmacologic treatment, a large proportion of patients can achieve symptom control and a low risk of adverse events, such as acute exacerbations. Treatment with inhaled corticosteroids (ICS) reduces symptoms, improves pulmonary function parameters such as forced expiratory volume in 1 s (FEV_1_), reduces risk of future exacerbations and asthma-related mortality [4–6]. However, adherence to controller medication, including ICS, is highly variable between asthma patients and is estimated to range between 22 to 70% of prescribed doses across different settings [2, 7, 8]. Possible reasons for non-adherence to asthma controller therapy are numerous and are often related to lack of perceived need for treatment and/or either fear of side effects or perceived side effects of ICS treatment [9]. However, not all reasons for non-adherence are based on personal beliefs or even intentional, as social factors outside of the patient’s own control, forgetfulness and misunderstandings have been shown to be significant contributors to non-adherence [7].

Low adherence to controller medication is associated with poor disease control and adverse outcomes in asthma. These include higher symptom burden, lower FEV_1_ and higher risk of hospitalization-requiring exacerbations [2]. As such, it is vital to address poor adherence, even though the complexity of factors associated with reduced adherence is daunting to clinicians and patients are reluctant to admit non-adherence [10]. Nonetheless, patients must be engaged in a discussion during consultations, as improving adherence requires a multimodal approach and establishing a partnership with patients [11]. To this end, clinically reliable tools for quick adherence assessments, both objective and subjective, are needed.

Adherence can be estimated objectively using several methods with electronic dose metering being the current gold standard for ICS adherence measurement [12, 13]. The Medication Possession Ratio (MPR), based on prescription data, is another method for measuring objective adherence, one that does not require access to specialized inhaler equipment. With regard to patient-reported adherence, the Danish Society of Respiratory Medicine’s Guidelines on Possible Severe Asthma recommends the systematic use of Foster score when assessing adherence [14]. The Foster score was developed as a simple, non-confrontational, one question scoring method for quick assessment based on the Morisky medication-taking behavior scale [9, 15]. However, its clinical value and relationship to objective adherence measurement remains unclear.

In the present study, we aimed to investigate the correlation between patient reported adherence assessed by Foster score and the objectively measured MPR in a university hospital asthma outpatient clinic.

## Methods

### Design, Study Population & Participant Enrolment

This study is a cross-sectional observational study carried out in the Respiratory Outpatient Clinic, Department of Respiratory Medicine, Copenhagen University Hospital -  Hvidovre, Hvidovre, Copenhagen, Denmark.

All patients with a routine follow-up appointment between January and June 2020 at the Respiratory Outpatient Clinic with an active asthma ICD-10 code (DJ45) were screened for inclusion using electronic patient medical records. Cohort inclusion required the following criteria: i) objectively confirmed asthma for at least 12 months prior to inclusion ii) at least 18 years of age. Exclusion criteria were as follows: i) patients prescribed ICS for less than 12 months at the index date, ii) recipients of dose dispensed medications via assisted care, iii) the inability to answer the questionnaire due to any psychological or physical limitations and iv) non-Danish residents without a Danish civil registration number and corresponding Common Medication Card.

### Ethics

The present study was approved by the Danish Patient Safety Authority (ref. 31–1521-118) and the Capital Region of Copenhagen’s Data Monitoring Board (ref. P-2020-648).

### Data collection

Data for age, sex, body mass index (BMI), Fractional Exhaled Nitric Oxide (FeNO), lung function parameters (FEV_1_, FEV_1_%pred, Forced Vital Capacity (FVC) and FVC%pred) and Asthma Control Questionnaire 6-score (ACQ6) was collected through electronic medical records (Sundhedsplatformen, Epic Systems Inc., USA).

Asthma exacerbation data was collected from electronic patient journals using the following definitions: i) Moderate exacerbation – either prescription of at least 37.5 mg oral prednisolone for at least 3 days not coinciding with a hospitalization, or hospitalization/emergency room admittance for less than 24 h. ii) Severe exacerbation – any exacerbation requiring hospitalization for at least 24 h and administration of oral or intravenous corticosteroids.

### Spirometry

Lung function was measured using a Pneumotrac (Vitalograph Ltd., Buckinghamshire, UK) spirometer as part of standard of care at the respiratory outpatient clinic.

### Allergies

Patients were considered to have allergic disease when 1) relevant symptoms and 2) relevant diagnostic workup (either positive skin prick test, blood samples positive for elevated specific Immunoglobulin E (IgE) or elevated total-IgE) were described in the electronic patient records.

### Prescription data

Pharmacy redemption data was collected for each patient from the national Common Medication Card, the national prescription register for the tax-funded universal healthcare insurance in Denmark. ICS, long-acting β2-agonist (LABA), long-acting muscarinic receptor antagonist (LAMA), leukotriene-receptor antagonist (LTRA), short-acting β2-agonist (SABA), theophylline, biologic therapy (anti-IL5(Ra), anti-IL4/13 and anti-IgE), oral corticosteroid (OCS) doses and proton-pump inhibitor (PPI) use was registered for 12 months prior to the index date.

Daily prescribed ICS dose was categorized according to the GINA 2020 guidelines [6]. Objective controller medication adherence (MPR) was calculated as the number of physician prescribed doses during the previous 12 months, divided by the number of redeemed doses during the previous 12 months [16]. During MPR calculations, the following adjustment were performed i) Patients prescribed two ICS inhalers were considered to be adherent to treatment if both doses were available for use at the same time. ii) For patients with dual ICS therapy prescriptions, only redeeming one of the ICS during the observation period, MPR was calculated as single ICS MPR for the inhaler redeemed and reduced by 50% to reflect non-adherence. iii) Patients changing inhalers during the observation period had the remainder of doses in the discontinued inhaler at the date of discontinuation discarded to prevent inflation of MPR.

### Patient reported adherence

Ten modifiable patient beliefs or behaviors regarding adherence to ICS has been identified by Foster and colleagues [9]. Based on the results of Foster et al. [9], a so-called Foster score has been adapted and recommended by the Danish Society of Respiratory Medicine Guidelines using the question *“How many days in a 7-day week do you take your medication as prescribed?*”. The Foster score is achieved by dividing the patients’ answer by seven and multiplying by 100. The resulting Foster score ranges from 0% (completely non-adherent) to 100% (fully adherent).

### Inhaler technique

All patients enrolled had inhaler technique, including inspiratory flow and device handling, assessed at both the index visit as well as any prior visits to ensure proper inhaler use.

### Statistics

Descriptive statistics were used to generate demographic data, with the results presented as means with standard deviations (SD). Groupwise comparisons, including non-responder analyses, were performed using either t-tests or Wilcoxon rank-sum tests, depending on the distribution of the data.

Non-responder analyses were performed, comparing demographics and asthma-related parameters of patients who provided clinicians with a Fosters score to those whose Foster scores were not recorded in their patient records.

Bivariate and multivariable linear regressions were performed to investigate the association between Foster score and MPR. Multivariable linear regression analyses were adjusted for age, FEV_1_, sex, GINA 2020 treatment step [6] number of hospitalizations due to asthma the past 24 months, prescribed oral corticosteroids and SABA overuse (defined as ≥600 doses per year).

Explorative multivariable linear regression was performed to assess the influence of common comorbidities and subjective disease control on Foster scores and ICS MPR. The model was adjusted for age, sex, ACQ6, BMI, GERD (Gastroesophageal reflux disease, defined as PPI-use at the index visit) and allergy. A *p*-value of 0.05 was considered as statistically significant.

R 4.0.0 (The R Foundation for Statistical Computing) was used for statistical analysis and to generate figures.

## Results

A total of 400 patients were screened in the respiratory outpatient clinic during the inclusion period, of which 313 were eligible for the study. Out of the 313 eligible patients, 135 patients failed to supply Foster scores during consultations and were thus excluded. The final cohort comprised of 178 patients, of whom 136 (76.4%) were female, and the mean age was 47 (SD 16) (Table 1).

**Table 1:** Baseline characteristics of the study sample comprising 178 patients with asthma managed at a university hospital respiratory outpatient clinic

Baseline Characteristics	N (%) or Mean (SD)
**Age (yrs.)**	47 (16)
**Female**	136 (76.4%)
**ACQ6-score**	1.32 (1.07, *N* = 114)
**FeNO**	20.3 (18.8, *N* = 164)
**BMI**	27.6 (5.8, *N* = 176)
**Allergic Disease**	59 (36.7%, *N =* 164)
**GERD**	41 (23.0%)
**FEV**_**1**_ **(L)**	2.79 L (0.80)
**FEV**_**1**_**%pred**	91% (18%)
**FVC (L)**	3.67 L (1.02, *N =* 169)
**FVC%pred**	102% (20, *N =* 169)
**FEV**_**1**_**/FVC**	0.76 (0.09, *N =* 169)
History of Moderate Exacerbations*	43 (24.2%)
24-month Moderate Exacerbation Rate	1.77 (1.27)
History of Severe Exacerbations**	23 (12.9%)
24-month Severe Exacerbation Rate	1.65 (1.50)
**GINA 2020 Asthma Severity Grade**	
Mild Disease	15 (8.4%)
Moderate Disease	60 (33.7%)
Severe Disease	103 (57.9%)

*defined as either prescription of at least 37.5 mg oral prednisolone for at least 3 days, or hospitalization/emergency room admittance for less than 24 h in the last 24 months prior to inclusion. **defined as any exacerbation requiring hospitalization for at least 24 h and administration of oral or intravenous corticosteroids. *N* number of patients, *yrs.* years, *ACQ* Asthma Control Questionnaire, *BMI* Body Mass Index, *FeNO* Fractional Exhaled Nitric Oxide, *FEV*_*1*_ Forced Expired Volume in the first second, *GERD* Gastroesophageal Reflux Disease, *GINA* Global Initiative for Asthma

### Lung function, comorbidities and asthma severity

In the final cohort, mean FEV1 was 91%pred (SD 18), corresponding to 2.79 L (SD 0.80) (Table 1). Mean BMI and FeNO were 27.6 (SD 5.8) and 20.3 ppb (SD 18.9), respectively. Fifty-nine patients (36.7%) were classified as having allergic disease and 41 patients (23.0%) were prescribed PPI for GERD.

When classified according to GINA 2020 severity 15 (8.4%), 60 (33.7%) and 103 (57.9%) were classified as mild, moderate and severe, respectively (Table 1). Regarding ICS doses, medium and high doses were prescribed to 71 (39.9%) and 41 (23.0%) patients, respectively (Table 2).

**Table 2:** Treatment regimens and patient-reported and objectively measured asthma medication adherence in 178 patients attending a university hospital outpatient clinic

Treatment and Adherence	N (%), Mean (SD)
**ICS Prescribed Dose**	
Low Dose	66 (37.1%)
Moderate Dose	71 (39.9%)
High Dose	41 (23.0%)
Dual ICS Therapy	32 (18.0%)
**GINA 2020 Step**	
Step 2	15 (8.4%)
Step 3	60 (33.7%)
Step 4	62 (34.8%)
Step 5	41 (23.0%)
**Biologic Therapy**	3 (1.7%)
**Maintenance Oral Corticosteroids**	6 (3.4%)
**Annual SABA Use (Doses)**	255 (445)
of which > 600 doses/yr	32 (18.0%)
**Foster Score**	94.0% (19.0%)
of which 100%	156 (87.6%)
**Inhaled Corticosteroid MPR**	0.54 (0.25)
Once-daily ICS MPR (*n* = 19)	67.0% (13.1%)
Twice-daily ICS MPR (*n* = 127)	53.4% (25.2%, *p* = 0.05*)
Dual ICS MPR (*n* = 32)	43.9% (26.0%, *p* = 0.02*)
**ICS Adherence**	
80% or above	30 (16.9%)
Below 80%	148 (83.1%)

* Versus patients receiving once-daily ICS. *N* number of patients, *IQR* inter-quartile range, *ICS* Inhaled Corticosteroids, *GINA* Global Initiative for Asthma (2020), *MPR* medication possession ratio, *LABA* long-acting β2-agonist, *LAMA* long-acting muscarinic receptor antagonist, *SABA* short-acting β2-agonist, *MPR* medication possession ratio, *SD* standard deviation

### Asthma control

In terms of disease control, 43 (24.2%) patients had had moderate exacerbations during the past 24 months. For patients with at least one moderate exacerbation, the mean 24-month moderate exacerbation rate was 1.77 (SD 1.27).

Of the included patients, 23 had been hospitalized due to an asthma exacerbation with a mean 24-month hospitalization rate of 1.65 (SD 1.50).

The mean annual SABA use was 255 (SD 445) doses/year, with 18.0% of patients defined as having an excessive SABA use above 600 doses/year (Table 2).

### Adherence measurements

In terms of patient-assessed adherence, the mean reported Foster score was 94.0% (SD 19.0%) with 156 (87.6%) patients reporting the best possible Foster score. Corresponding objective adherence was a mean ICS MPR of 54.0% (SD 25.0%), with 16.9% of patients deemed as being objectively adherent (ICS MPR above 80%).

When stratified by ICS treatment regimen as once-daily, twice-daily or dual ICS, the mean ICS MPR was significantly lower in both twice-daily and dual ICS-regimens when compared to patients receiving once-daily ICS-formulations (Table 2).

### Correlation between MPR and patient reported adherence

**Fig. 1:**
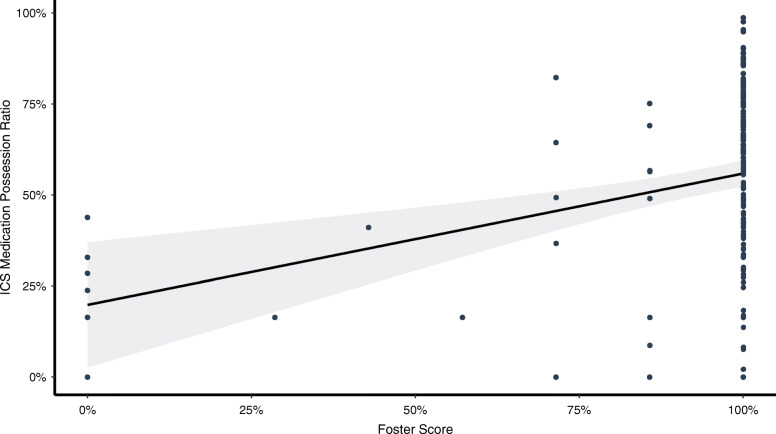
Distribution of ICS MPR and Foster scores for 178 patients attending a university hospital respiratory outpatient clinic, as well as a linear regression on the relationship between ICS MPR and Foster scores. *R*^2^ = 0.082

In bivariate regression analysis, a significant association between MPR and Foster score was demonstrated, with an absolute 36% MPR difference (Beta 0.36, 95% CI 0.18–0.54; *p* < 0.001) between patients reporting Foster scores 0 and 100%. However, the model fit was poor (*R*^2^ 0.082), even though the overall model demonstrated a significant *p*-value of < 0.001 (Fig. 1).

In multivariable analysis adjusted for age, FEV_1_, sex, GINA 2020 treatment step, history of moderate and severe exacerbations in the past 24 months and excessive SABA use, Foster score remained statistically significantly associated with ICS MPR, with an absolute MPR increase of 32% between patients reporting a Foster score of 0 and 100% (Beta 0.32, 95% CI 0.13–0.50; *p* < 0.001). Female sex was associated with a higher ICR MPR (0.12, 95% CI 0.02–0.22; *p* = 0.019) while a history of severe exacerbations the past 24 months was associated with a lower ICS MPR (− 0.12, 95% CI -0.24 - -0.01, *p* = 0.039). Additionally, a trend towards a higher MPR with increasing FEV_1_ was found (0.05, 95% CI -0.01 - 0.11, p 0.079). The overall model achieved an adjusted *R*^2^ of 0.149 with a corresponding *p*-value of 0.001 (Table 3).

**Table 3:** Bivariate and multivariable linear regressions with ICS MPR as outcome variable and Foster score, age, FEV_1_, sex, GINA 2020 treatment steps, exacerbation history and excessive SABA use as exposure variables

Variable	Bivariate			Multivariable		
	Beta	95% CI	***P***-value	Beta	95% CI	***P***-value
**Foster Score**	0.36	(0.18–0.54)	< 0.001	0.32	(0.13–0.50)	< 0.001
**Age**				0.00	(0.00–0.00)	0.2
**FEV**_**1**_				0.05	(-0.01–0.11)	0.079
**Female**				0.12	(0.02–0.22)	0.019
**GINA 2020 Step**						
Step 2				–	–	–
Step 3				0.02	(-0.11–0.16)	0.8
Step 4				0.10	(-0.04–0.24)	0.2
Step 5				0.08	(-0.08–0.24)	0.3
**History of Moderate Exacerbations**^#^				0.00	(-0.09–0.10)	> 0.9
**History of Severe Exacerbations**^##^				-0.12	(-0.24–-0.01)	0.039
**Excessive SABA use (above 600 doses/yr)**				0.06	(-0.04–0.16)	0.3

*R*^2^ = 0.082. Adjusted *R*^2^ = 0.149. *FEV*_1_ Forced Expired Volume in the first second, *GINA* Global Initiative for Asthma (2020), ^#^defined as either prescription of at least 37.5 mg oral prednisolone for at least 3 days, or hospitalization/emergency room admittance for less than 24 h in the last 24 months prior to inclusion. ^##^defined as any exacerbation requiring hospitalization for at least 24 h and administration of oral or intravenous corticosteroids, *SABA* Short-acting beta 2-agonist

### Explorative analyses

In multivariable linear regression adjusted to investigate correlation between ICS MPR, Foster score and the role of comorbidities and patient-reported disease control, Foster score remained associated with ICS MPR though with a neutral beta (0.00, 95% CI 0.00, 0.01; *p* = 0.006). Of the chosen covariates (age, sex, ACQ6, BMI, GERD and allergic disease) only GERD was significantly associated with ICS MPR (Beta − 0.13, 95% CI -0.25, − 0.01; *p* = 0.040) (Table 4).

**Table 4:** Multivariable linear regression with ICS MPR as outcome variable and Foster score, age, sex, ACQ6 and common comorbidities (BMI, GERD and allergies) as exposure variables

Variable	Multivariable		
	Beta	95% CI	***P***-value
**Foster Score**	0.00	(0.00–0.01)	0.006
**Age**	0.00	(0.00–0.01)	0.061
**Female**	0.04	(-0.07–0.15)	0.4
**ACQ6**	0.01	(-0.04–0.05)	0.8
**BMI**	0.00	(-0.01–0.01)	0.6
**GERD**	−0.13	(-0.25–0.01)	0.040
**Allergy**	0.06	-0.05–0.17	0.3

Adjusted *R*^2^ = 0.155. *ACQ6* Asthma Control Questionnaire 6, *BMI* Body Mass Index, *GERD* Gastroesophageal reflux (Proton-pump inhibitor-use), Allergy (Relevant symptoms plus either positive skin prick test or elevated specific Immunoglobulin E)

### Non-responder analyses

Of patients screened for inclusion, participating patients were more likely to be younger, have a slightly lower ACQ and higher lung function variables. However, no differences in objective disease control measurements or adherence to ICS were found (Supplementary Table 1).

## Discussion

This study sought to investigate the relationship between patient-reported adherence and objective adherence measured by MPR for the past 12 months. Patient-reported adherence was high with a mean of 94.0% and was significantly associated to ICS MPR, though demonstrating a weak direct correlation with overall MPR scores.

We have shown a high prevalence of patients reporting perfect adherence with a Foster score of 100% despite high MPR variance (MPR range 0.0–99.7%). Patient overestimation of adherence is probable, as a significant portion of patients who reported a Foster score of 100% did not have a corresponding MPR above 80%. Indeed, patient-assessed adherence is vulnerable to social desirability bias and unintentional non-adherence. In accordance with our findings, several studies have found patient-reporting to inflate ICS adherence rates by 30–90% [17–19] and similar effects are seen in other chronic diseases [20, 21].

For a patient to be considered adherent, a MPR threshold of 80% or higher is typically required [2, 22, 23]. In the present study, objectively measured mean adherence (MPR) was in line with previous studies ranging from 8 to 70% [2, 19, 24, 25]. However, we observed a disparity between the number of patients reporting perfect adherence using Foster score (87.6%) and objectively measured acceptable (MPR ≥80%) adherence (16.9%). Such discrepancies are well described in the literature [17], signaling a difference in what is deemed an acceptable level of medication use between clinicians and patients, while highlighting the importance of establishing a partnership with patients and engaging in deeper conversations regarding the use of ICS.

The high prevalence of perfect adherence reported by patients challenges the use of correlation and regression statistics due to high homogeneity and resulting lack of variance and highlights a major weakness in clinical use of the Foster score and limits applicability. However, both regression models demonstrated significant, yet weak, relationships between ICS MPR and Foster score and explained variability measured by *R*^2^ values were low, reaching approximately 15% in the multivariable model. Foster and colleagues previously investigated the relationship between ICS-related beliefs and MPR and demonstrated that an 11-item model explained two thirds of objective adherence to ICS [9], suggesting that psychological factors represent a larger portion of overall adherence-deciding factors than clinical parameters used in the present study. Other studies have demonstrated that other patient-specific factors, such as socioeconomic status and comorbidity burden, have profound effects on adherence [7, 26]. Indeed, clinical adherence assessment should include multiple domains, both objective and subjective. Such tools are in continuous development, and the Test of Adherence to Inhalers is a validated example of a multi-domain adherence assessment tool [27].

In regression analyses, a history of previous severe exacerbations within the last 24 months was significantly associated with lower adherence. In contrast to a history of severe exacerbations, a history of moderate exacerbations within the last 24 months was not significantly associated with adherence. This suggests that within this population a higher adherence primarily protects against severe exacerbations. The protective effect of ICS adherence on severe exacerbations has been well established [2, 28], but recent results and differences in adherence and exacerbation definitions cast doubt on the relationship between adherence, exacerbation history and the risk of future severe, hospitalization-requiring exacerbations [29].

Interestingly, our data suggests that practical factors are associated with adherence, as patients with less complex treatment regimes, such as once-daily dosage, demonstrate a higher MPR than those with twice-daily dosing. Furthermore, the use of more than one ICS inhaler was associated with a lower MPR. This has previously been reported in a plethora of chronic diseases treated with oral medications, showing that one pill taken once-daily is associated with the highest adherence [30]. As such, practical factors are important pieces in the adherence puzzle beyond classic psychological and clinical factors. As such, clinicians should not only take beliefs and disease severity into account when prescribing inhaler treatments, but also gauge individual patients’ ability to follow complex regimens.

There are several caveats to using MPR as a measurement of objective adherence. First, the Foster score is a time-agnostic measurement, while MPR always takes a retrospective long-term perspective, demonstrated in the present article by 5 patients reporting a Foster score of 0 while having an MPR above 0. Second, depending on the chosen methodology MPR measurements are sensitive to inflation when generated from pharmacy records [31]. MPR inflation can occur due to changes in inhaler therapy shortly after treatment initiation, unadjusted dual-ICS therapy (such as a combination of moderate doses of fluticasone and ciclesonide) and redemption of multiple ICS inhalers to use as backup. However, in the present study, MPR inflation has been mitigated in part due to adjusting for inhaler changes by removing excess doses after the physician-ordered discontinuation date, dual-ICS adjustment as well as MPR capping. The use of MPR capping has been thoroughly discussed, with previous studies reporting limited significance of capping MPR, especially in contrast to other adherence measurements such as proportion of days covered [9, 32].

### Limitations

Several limitations exist in this study. First, the study covers a set of objective and subjective adherence measurements, reducing generalization to other methods of assessing adherence. Second, while prescription data are accurate in measuring the number of doses redeemed, there is a risk of redeemed doses remaining unused by the patient either due to low adherence or due to a change in medication regimens. Poor inhaler technique may lead to administered but theoretically lower doses which cannot be assessed using MPR, though all outpatients are provided with inhaler training as part of every outpatient visit at the study center. Due to the study design, patients redeeming inhalers in bulk may be either under- or overestimated in terms of number of redeemed doses, should the patient have redeemed multiple prescriptions just outside the study period. Prescription auto-refills are unavailable in Denmark, but have previously been shown to affect adherence with long-term medications and external validity may thus be limited [33]. Furthermore, the cohort has a high prevalence of female participants increasing the risk of bias, though the role of sex in adherence is debated [34] and sex is adjusted for in multivariable analyses. Finally, slight differences between responders and non-responders was found, however with a large span and with most variables below clinical differences. Outcome measurements such as objective disease control and MPR were not found to be differing between responders and non-responders.

## Conclusion

While a significant association between Foster score and medication possession ratio with inhaled corticosteroids was found, our findings do not support the use of Foster score as the sole marker of adherence with controller medication in asthma. Future studies should address the complex interplay between self-reported and objectively assessed adherence with controller medication in asthma. 

## Supplementary Information

**Additional file 1: Supplementary Table 1.** Non-responder analyses of baseline and disease control charateristics of 313 patients eligible for inclusion.

## Data Availability

Data is available upon reasonable request. However, approval from data sources and the Capital Region of Copenhagen’s Data Safety Board may be required as per Danish law.
