# Supplementary material for: Self-reported vs. objectively assessed adherence to inhaled corticosteroids in asthma
Source: Asthma Res Pract. 2021 May 31;7:7. doi: 10.1186/s40733-021-00072-2 (PMC8166004; doi:10.1186/s40733-021-00072-2)
Supplement: Supplementary file 1 — Additional file 1: Supplementary Table 1. Non-responder analyses of baseline and disease control charateristics of 313 patients eligible for inclusion. [file 40733_2021_72_MOESM1_ESM.docx]

**SUPPLEMENTARY MATERIALS**

**Self-reported vs. objectively assessed adherence to inhaled corticosteroids in asthma**

**Supplementary Table 1**

Non-responder analysis of 313 patients eligible for inclusion in the present study.

| Baseline Characteristics | Non-responders | Responders | P-value |
| --- | --- | --- | --- |
| Age (yrs.) | 54 (16) | 47 (16) | <0.001 |
| Female | 98 (72.6%) | 136 (76.4%) | 0.4 |
| ACQ-score (n=230) | 1.62 (1.11) | 1.32 (1.07) | 0.031 |
| FEV_1_ (L) | 2.49 (0.84) | 2.79 (0.80) | 0.002 |
| FEV_1_%pred | 85 (18) | 91 (18) | 0.005 |
| FVC (L) | 3.37 (1.06) | 3.67 (1.02) | 0.008 |
| FVC%pred | 96 (18) | 102 (20) | 0.008 |
| FEV_1_/FVC | 0.73 (0.09) | 0.76 (0.09) | 0.050 |
| History of Moderate Exacerbations* | 36 (26.7%) | 43 (24.2%) | 0.13 |
| 24-month Moderate Exacerbation Rate | 1.00 [1.00;2.00] | 1.00 [1.00;2.00] | 0.8 |
| History of Severe Exacerbations** | 15 (11.1%) | 24 (13.5%) | 0.2 |
| 24-month Severe Exacerbation Rate (Median, IQR) | 1.00 [1.00;2.00] | 1.00 [1.00;1.00] | 0.4 |
|  |  |  |  |
| ICS Prescribed Dose |  |  | 0.002 |
| Low | 36 (26.667%) | 66 (37.079%) |  |
| Medium | 81 (60.000%) | 71 (39.888%) |  |
| High | 18 (13.333%) | 41 (23.034%) |  |
| GINA 2020 Step |  |  | 0.006 |
| Step 2 | 9 (6.667%) | 15 (8.427%) |  |
| Step 3 | 35 (25.926%) | 60 (33.708%) |  |
| Step 4 | 73 (54.074%) | 62 (34.831%) |  |
| Step 5 | 18 (13.333%) | 41 (23.034%) |  |
| Yearly SABA Use (Doses) | 253 (545) | 255 (445) | 0.8 |
| of which >600 doses/yr | 15 (11.111%) | 32 (17.978%) | 0.092 |
| ICS Adherence | 0.51 (0.28) | 0.54 (0.25) | 0.4 |
| 80% or above | 19 (14.074%) | 30 (16.854%) | 0.5 |

*defined as either prescription of at least 37.5 mg oral prednisolone for at least 3 days, or hospitalization/emergency room admittance for less than 24 hours in the last 24 months prior to inclusion. **defined as any exacerbation requiring hospitalization for at least 24 hours and administration of oral or intravenous corticosteroids. N = number of patients, yrs. = years, ACQ = Asthma Control Questionnaire, FEV_1_ = Forced Expired Volume in the first second.
